# Contribution of Oxygen and Ultraviolet Light to the Adhesion Properties of Warm Mix Asphalt During the Light-Oxidative Coupling Ultraviolet Aging Process

**DOI:** 10.3390/ma18061345

**Published:** 2025-03-18

**Authors:** Jianbing Ma, Bo Li, Yongning Wang, Xiaomin Li, Dongna Li, Xiaoyu Ren, Mingxing Fu

**Affiliations:** 1 Gansu Province Traffic Investment Management Co., Ltd., Lanzhou 730030, China; majb2004@126.com; 2 National and Provincial Joint Engineering Laboratory of Road & Bridge Disaster Prevention and Control, Lanzhou Jiaotong University, Lanzhou 730070, China; lidongna@mail.lzjtu.cn; 3 Gansu Province Transportation Planning, Survey & Designing Institute Co., Ltd., Lanzhou 730030, China; 13919772255@139.com; 4 School of Highway and Construction Engineering, Yunnan Communications Vocational and Technical College, Kunming 650500, China; 18811016172@163.com; 5 China Energy Construction Group Gansu Electric Power Design Institute Co., Ltd., Lanzhou 730070, China; 19893176505@163.com

**Keywords:** warm mix asphalt, ultraviolet aging, adhesive performance, functional groups, contribution rate

## Abstract

Objective: We investigated the contribution of oxygen and UV light to the UV aging process of warm mix asphalt (WMA). Methods: In this paper, warm mix asphalt was prepared with different aging methods (RTFOT, PAV and UV) and UV aging times (50 h, 100 h, 150 h and 200 h). The cohesion and bonding functions of WMA were tested using surface free energy theory. In addition, the UV aging functional groups of WMA were analyzed using Fourier transform infrared spectroscopy (FTIR). On this basis, the contribution of oxygen and ultraviolet light to the UV aging of WMA was analyzed using the random forest model. Results and conclusions: The results showed that UV aging had the greatest effect on the adhesion property index and functional group index of WMA, followed by PAV aging, and RTFOT aging had the least effect. With the extension of UV aging time, the adhesion and cohesion functions of WMA showed a decreasing trend, while the carbonyl index and sulfoxide index showed an increasing trend. When the UV aging time exceeded 150 h, the adhesion function and functional group index of WMA gradually tended to stabilize. The effect of UV aging on the adhesive properties of WMA was mainly due to adhesive damage. There were significant differences in the effects of oxygen isolation and light–oxygen-coupled UV aging on the adhesive properties and functional group index of WMA. In the light–oxygen-coupled UV aging of warm mix asphalt, the contribution of UV radiation was 79.9%, and the contribution of oxygen was 20.1%.

## 1. Introduction

Asphalt pavements are widely used in the construction of highways of different grades due to their good properties [1,2]. Asphalt pavement consists of asphalt, aggregate, filler and voids. The asphalt binding material plays an important role in bonding the aggregates and fillers to give the pavement better bearing capacity and driving comfort [3]. However, asphalt is susceptible to aging under the influence of temperature, light, oxygen, water and other factors during long-term use [4,5,6]. A series of complex physicochemical reactions continuously occur in the aging process of asphalt. The high-temperature, low-temperature and adhesion properties of asphalt are significantly affected. Therefore, accurately simulating the real environmental conditions of indoor aging acceleration and aging evaluation methods are current research hotspots.

Asphalt aging mainly consists of thermo-oxidative aging and UV aging [7,8]. In the past decades, the thermo-oxidative aging process of asphalt has been evaluated mainly by means of the thin-film oven test (TFOT), rolling film oven test (RTFOT) and pressurized aging vessel (PAV). TFOT and RTFOT aging simulate the aging process of asphalt from the mixing of asphalt mixtures to the on-site paving of asphalt pavements. PAV aging simulates the aging of asphalt from the on-site paving of asphalt mixtures to the end of the service life of the pavements [9,10,11]. The results of Diouri et al. showed that cracks in asphalt pavements increase with the degree of aging, and this change is more significant with increasing temperature [12]. Li et al. showed that as asphalt binders age, colloidal particles aggregate, which affects changes in their basic physicochemical properties. These changes translate into changes in the engineering properties of asphalt binders [13]. Liu et al. concluded that volatilization of asphalt binding material components, oxidation reactions and migration of components are key factors in determining the degree of aging [14]. Wang et al. found that the high temperature performance increased with the degree of aging. After aging, the irrecoverable creep compliance decreased, and the creep recovery percentage increased significantly [15]. Previous studies have found that the current research on thermo-oxidative aging of asphalt is basically mature. The factors affecting thermo-oxidative aging have been basically elucidated, and the mechanism of thermo-oxidative aging is basically clear. However, there is a lack of indoor simulation devices and characterization methods for UV aging, one of the key factors affecting asphalt aging.

To solve the indoor simulation device problem of asphalt UV aging, researchers simulated the effects of UV, oxygen and temperature on the performance of asphalt pavement and developed corresponding UV aging test devices [16,17,18]. Initially, Montepara et al. independently developed a UV aging test chamber. UV aging was conducted using a mercury lamp at a temperature of 140 °C, with an asphalt sample film thickness of 1.5 mm. Different degrees of aging were simulated for actual asphalt pavement at 1 year, 2 years, 6 years and 10 years [19,20]. However, due to the high experimental temperature, the results were significantly different from the actual situation. Subsequently, Bocci and Cemi designed a simulation method for UV-accelerated asphalt aging and found that UV aging is completely different from thermal oxidation aging [21]. Ye et al. established a simulation system for asphalt UV aging, further proving that light–oxidative aging is completely different from thermal oxidative aging [22]. On this basis, Pang et al. improved the UV aging test method from the perspective of selecting UV aging light sources and temperature control [23]. Zeng et al. proposed suggestions for selecting the thickness of UV-aged asphalt coatings [24]. Li et al. provided relevant references for the wavelength selection of ultraviolet aging [25].

Meanwhile, scholars have conducted a lot of research on the performance change and aging mechanism of asphalt after UV aging through indoor simulation devices. Xiao et al. used Materials Studio software 2020 to simulate the dynamic behavior of asphalt microstructure during UV aging, showing that with the progress of UV aging, the light components of asphalt molecules gradually transformed into heavy components [26]. Zhang et al. found that the carbonyl index increases with the increase in the UV aging degree of the asphalt binder, and when the asphalt binder tends to the aging limit, the carbonyl index also tends to be stable [27]. Zeng explored that the asphalt aging index and the deterioration performance of the SBS modifier increased significantly at a temperature of 70 °C, and the mass loss of asphalt after UV aging was four times that of thermal aging conditions [28]. Jamal et al. compared thermal aging and UV aging and found that thermal aging makes the light components volatilize, and the aliphatic index decreases. However, UV aging mainly contains aliphatic hydrocarbon chain components whose volatile bond energy is lower than 413 kJ/mol, such as C-C, C-O and C-H [29]. Rajib’s research shows that UV aging is more likely to deteriorate the asphalt performance than thermal oxygen aging, and the asphalt binder containing biochar can delay the UV aging process [30]. Liu et al. found that two processes may exist during the UV aging process of asphalt, namely the molecular excitation process and the chemical bond breaking process [31].To sum up, the UV aging process of asphalt is relatively complex, and scholars have focused on considering the evolution law of asphalt road performance under the joint action of UV and oxygen and initially analyzed the UV aging mechanism of asphalt. However, none of the current studies have considered the single contribution of oxygen and UV radiation in the UV aging process.

In addition, warm mix asphalt (WMA) is used as a green road construction material. Scholars have conducted extensive research on the effects of its various warm mix mechanisms on road performance and its aging performance evolution during long-term service [32,33,34,35,36]. The adhesion performance of WMA asphalt after UV aging, as a key indicator affecting its service life, has not yet been studied for the evolution law of adhesion performance during the UV aging process of WMA. Meanwhile, the contribution rate of oxygen and ultraviolet radiation to the adhesion performance of WMA during UV aging is not yet clear, which restricts its promotion and application in engineering.

We investigate the evolution pattern of the adhesion properties of WMA after photo-oxidative coupling and oxygen-insulating UV aging, as well as the contribution of oxygen to the effect of its UV aging adhesion properties. In this paper, two UV aging methods, oxygen isolation and light–oxygen coupling, were designed to carry out the UV aging of WMA. WMA samples with different aging methods (RTFOT, PAV and UV) and UV aging times (50 h, 100 h, 150 h and 200 h) were prepared. Then, the surface free energy theory was used to calculate the cohesive work and adhesive work of different samples. Based on the carbonyl and sulfoxide functional group index obtained by infrared spectroscopy, the relationship between the adhesive performance index and the functional group index was established. Then, significant difference analysis was used to compare and analyze the differences between oxygen barrier WMA and light–oxygen-coupled UV aging. Finally, a random forest model was used to analyze the contribution rates of oxygen and ultraviolet radiation in the light–oxidative coupling aging process of warm mix asphalt. The research results can provide some references for the establishment of asphalt ultraviolet aging evaluation methods and standards.

## 2. Materials and Methods

### 2.1. Experimental Design

**Figure 1:**
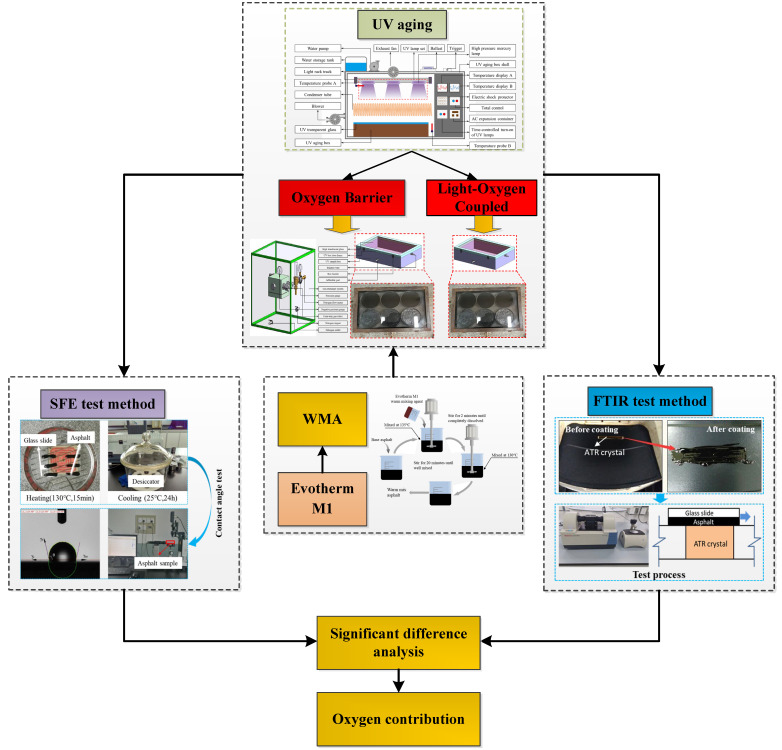
Flowchart of experimental design.

In this paper, two UV aging methods, oxygen isolation and photo-oxygen coupling, were used to prepare WMA aging samples, and the evolution law of adhesion property and the change law of functional group index of WMA aging were investigated by surface free energy theory and infrared spectroscopy testing. The contribution of oxygen to the UV aging adhesion properties of WMA was analyzed by a random forest model. Figure 1 depicts the experiment design flowchart for this investigation, together with the test procedures and raw materials used.

### 2.2. Experiment Design of UV Aging

#### 2.2.1. UV Aging Device Design

(1) UV aging environment device design

**Figure 2:**
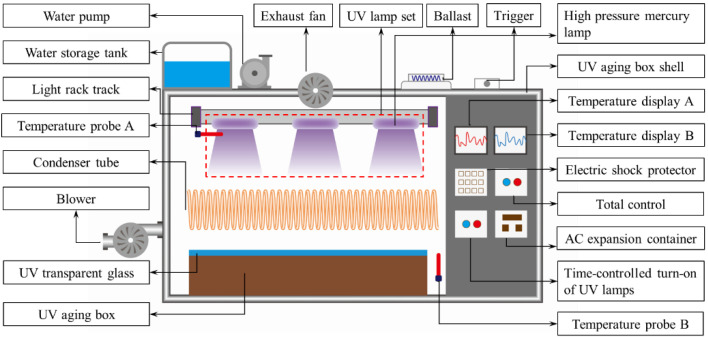
Rendering of UV aging device.

The independently developed ultraviolet aging chamber includes a high-pressure mercury lamp, blower, ventilation fan, sample box and temperature monitoring system. This time, the high-pressure mercury lamp model GYL230 was used as the light source for simulating asphalt UV aging, with a spectral range of 320–450 nm, a main peak value of 365 nm and a power of 250 W–22 KW, controlling its irradiation intensity at 200 w/m^2^. WMA was aged after RTFOT, it was UV-aged by a self-developed UV aging box, which the temperature controlled within the range of 60 °C~130 °C, and the UV intensity was controlled between 50 w/m^2^ and 600 w/m^2^. The UV aging equipment is shown in Figure 2.

(2) Design of oxygen insulation aging box

**Figure 3:**
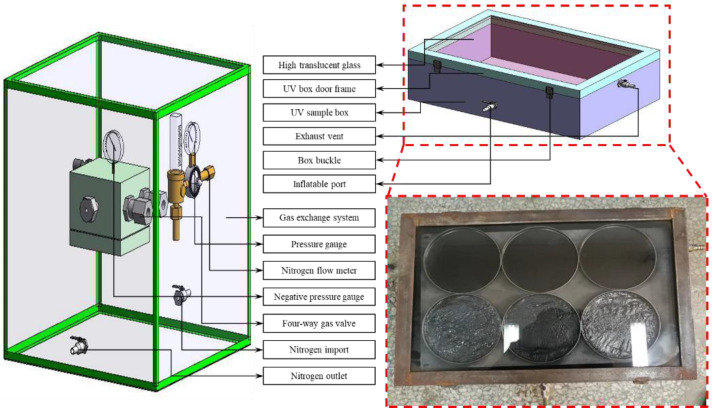
Oxygen insulation UV aging device.

The aging of asphalt is usually divided into single aging caused by factors such as light, heat, water and oxygen and multi-factor coupled aging. In order to study the aging of asphalt under single UV aging conditions, it is necessary to control the effects of factors such as heat, water and oxygen on the UV aging process of asphalt. For this purpose, a separate oxygen isolation aging chamber was designed to control the effect of oxygen on asphalt aging. In order to avoid the influence of oxygen on the experiment, a cubic aging chamber without a top cover was made of 1 cm-thick steel plate. The upper edge of the aging box was grooved and glued, and silicone rolls and UV transparent glass were placed. A thin wooden strip was placed above the glass and sealed with an iron cover (as shown in Figure 3). The specific steps are as follows:

a. Inject nitrogen into the oxygen-barrier aging box through the air inlet of the external nitrogen bottle, so that the air in the aging box is completely discharged.
b. Use ignition test at the air outlet to verify the oxygen concentration in the box.
c. After the gas exchange is over, close the air inlet and air outlet in time to avoid the entry of outside air.

#### 2.2.2. UV Aging Parameter Selection

**Figure 4:**
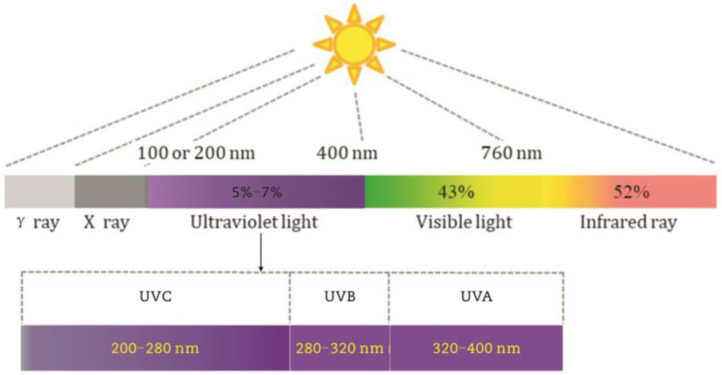
The distribution of solar spectrum.

Solar radiation is a very important factor causing road damage. The solar spectrum can be divided into ultraviolet (100–380 nm), visible (380–780 nm), near-infrared (780–2500 nm) and far-infrared (2500–60,000 nm) by wavelength. Among them, the wavelength of 100–280 nm is ultraviolet (UVC), the wavelength of 280–320 nm is ultraviolet (UVB), and the wavelength of 320–380 nm is ultraviolet (UVA) (see Figure 4). The ultraviolet radiation with a wavelength of 100–280 nanometers is the shortest and belongs to strong waves. Due to the strong absorption of ozone, ultraviolet radiation cannot reach the ground and will not cause damage to asphalt pavement. Ultraviolet rays with wavelengths ranging from 280 to 320 nanometers belong to the medium wave category and have strong effects. Most of them are absorbed by ozone, and the number of ultraviolet rays reaching the ground is relatively small. In the solar spectrum, ultraviolet energy with wavelengths of 320–380 nanometers is the highest and cannot be fully absorbed by ozone. Ultraviolet radiation ranging from 280 nanometers to 380 nanometers can damage the molecular structure of asphalt materials, leading to a decrease in the performance of asphalt pavement [24,25]. Therefore, it is necessary to choose a UV light source with a wavelength of 280~380 nm to simulate UV aging under natural conditions.

On the other hand, it is also necessary to consider the irradiation intensity of the light source. Usually, the UV aging of asphalt takes several months to find its aging pattern. If the light source intensity is too low, the simulation time will be too long, making it difficult to complete the simulation experiment [26]. Based on the conclusions drawn by current researchers [24,25,26], this article uses GYL230 high-pressure mercury lamp (made in China Zhuozhou Xupuri Electric Light Source Manufacture, Zhuozhou, China) as a light source to simulate asphalt UV aging. The spectral range is 320–450 nm, the main peak is 365 nm, the power is 250 W–22 KW, and the irradiation intensity is controlled at 200 w/m^2^.

#### 2.2.3. Determination of Laboratory-Simulated UV Aging Parameters

The ultraviolet radiation intensity of asphalt usually consists of three aspects: the total annual radiation under natural conditions, the radiation intensity of high-pressure mercury lamps, the distance of asphalt samples and the distance of high-pressure mercury lamps. The total annual ultraviolet radiation under natural conditions varies with altitude and latitude. Generally speaking, the western region is larger than the eastern region, and the ultraviolet radiation in high-altitude areas is stronger than that in low-altitude areas. Referring to the annual solar radiation in the western region of China, a maximum radiation of 7000 MJ/m^2^ was selected as the simulated total solar radiation. The proportion of ultraviolet radiation to total radiation is about 6% per year, which means the simulated annual total ultraviolet radiation is 420 MJ/m^2^.

When the asphalt sample is 23 cm away from the high-pressure mercury lamp, the surface temperature of the sample remains stable at 68 ± 3 °C, and the irradiation intensity remains stable at 200 ± 2 w/m^2^. Laboratory UV simulation time = total natural UV radiation/laboratory UV intensity. After conversion, the simulated one-year UV exposure time is 583 h. According to Equation (1), the indoor simulated UV aging time is set to 50 h, 100 h, 150 h and 200 h using time as a parameter for conversion [24]. The corresponding relationship with natural conditions is shown in Table 1.

(1) $$\frac{{420\;\mathrm{MJ}/m}^{2}{\times 10}^{6}}{{200\;W/m}^{2}}=2{.1\;\times \;10}^{6}(s)=583\;h$$

### 2.3. Sample Preparation

#### 2.3.1. WMA Samples

**Figure 5:**
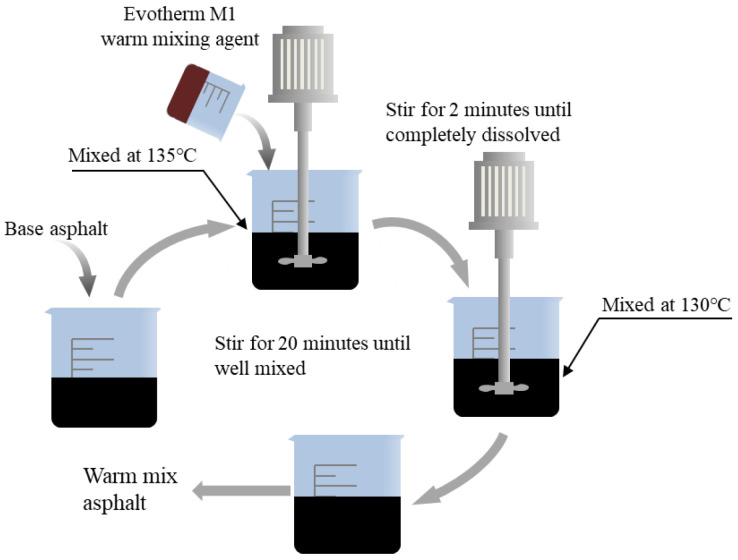
WMA preparation flowchart.

WMA was prepared as follows. First, the base asphalt was heated in an oven at 135 °C to a fluid state. Second, 500 g of base asphalt was weighed, to which the mass fractions of 0.5% and 1.0% Evotherm M1 were added and mixed evenly at low speed for 2 min with a mixer. Finally, the mixture was stirred for 20 min at 130 °C and 1200 r/min. Its preparation is shown in Figure 5. WMA was prepared from the neat #90 asphalt binder (graded based on the penetration value) and Evotherm M1. Its performance parameters are shown in Table 2. Base asphalt was provided by Gansu Province Transportation Planning, Survey & Designing Institute Co., Ltd. (Lanzhou, China). Evotherm M1 was provided by the Medvevik company, USA.

#### 2.3.2. Short-Term Aging Samples

An RTFOT was used to simulate the short-term aging process of 35 ± 0.5 g of WMA at 163 °C, with a turntable speed of 15 ± 0.2 r/min and air flow of 400 ± 200 mL/min for 85 min.

#### 2.3.3. Long-Term Aging Samples

After short-term aging of WMA, 50 ± 0.5 g of short-term aging samples was weighed for long-term aging. The aging temperature of PAV is 100 ± 0.5 °C, the pressure is 2.1 ± 0.1 MPa, and the aging time is 20 h.

#### 2.3.4. UV Aging Samples

The UV aging sample preparation process was as follows: ① 15.4 g of RTFOT aged asphalt was placed in a 14 cm diameter asphalt aging tray (asphalt film thickness of 1 mm) and placed in an oven at 163 °C for 10 min to prepare UV aging samples. ② The obtained samples were put into a homemade UV aging simulation system, and UV aging was performed at different times to simulate the light–oxidative aging of the asphalt pavement during its use. The main parameters of this test were as follows: temperature range of 65~70 °C and average irradiation intensity of 200 w/m^2^.

### 2.4. Test Methods

#### 2.4.1. Contact Angle Tests

The asphalt binder samples were prepared by heating at 163 °C and then poured onto small plates which were placed on a heater ahead of time to reach a constant temperature of 60 °C. The plates containing the asphalt binder were then heated with another heater at 163 °C for about 5 min to form a uniform film coating on the surface of the plates. Finally, the samples were cooled to room temperature and kept in a desiccator at room temperature for 12 h and then tested. A German OCA25 video optical contact angle meter was selected to measure the contact angle by titrating the contact angle samples with three known liquids by the prone drop method. Surface free energy parameters of three test liquids are shown in Table 3. In this case, the contact angle test evaluates the intrinsic interaction between the liquid and the solid, but the complex composition of tap water may introduce variables that cannot be controlled (e.g., calcium and magnesium ions reduce the surface tension, and organics form surface contamination), and for this reason distilled water was chosen for this paper.

#### 2.4.2. FTIR Tests

Infrared spectroscopy experiments were conducted using ATR crystal plates. The main experimental parameters include a scanning range of 400~4000 cm^−1^, scanning frequency of 32 times and minimum resolution of 0.019 cm^−1^. When performing the specific operation, the asphalt was applied onto the potassium bromide sheet of the infrared spectrum. Infrared spectral scanning can be performed according to the set testing parameters. It should be noted that the thickness of asphalt application has no effect on the test results, except when applying asphalt. To prevent asphalt aging, it is necessary to completely cover the asphalt with potassium bromide chips.

### 2.5. Surface Free Energy Theory

The SFE of a substance is the amount of energy required to produce a surface unit in that substance [37]. The literature [38] suggests a good correlation between the cohesion and adhesion work calculated from the surface free energy and the adhesion between the actual asphalt binding material and the aggregate. The SFE is made up of Lewis polar component ($\gamma^{AB}$) and Lifshitz nonpolar component ($\gamma^{LW}$), based on the Good-van Oss-Chaudhury hypothesis. The Keesom orientation component, Debye induced component and London dispersion component make up the Lifshitz nonpolar component, also known as the van der Waals component. The Lewis acid component ($\gamma^{+}$) and the Lewis base component ($\gamma^{-}$) make up the Lewis polar component, which is also known as the Lewis acid–base component [32,38]. In this sense, the SFE components provided in Equation (2) can be used to express the total SFE:

(2) $$\gamma =\gamma^{LW}+\gamma^{AB}=\gamma^{LW}+2\sqrt{\gamma^{+}\gamma^{-}}$$

#### 2.5.1. Cohesive Work

The energy needed to cause a material to crack and form two interfaces from one is known as the cohesive work [32]. This definition states that the asphalt binder’s cohesive work is represented by Equation (3) as follows:

(3) $$W_{a}=2\gamma_{a}$$

where $W_{a}$ is the cohesive work, and $\gamma_{a}$ is the total SFE of asphalt binder.

#### 2.5.2. Adhesive Work

Adhesion work refers to the bonding performance between asphalt and stone under anhydrous conditions, which is another major aspect of water damage in warm mix asphalt pavement. Limestone is a commonly used aggregate for asphalt pavement; therefore, this study selected limestone as the aggregate. The surface free energy of limestone used in this article is 219.9 mJ/m^2^, with a dispersed component of 51.9 mJ/m^2^ and a polar component of 168 mJ/m^2^. The adhesion work is calculated according to Equation (4). Under anhydrous conditions, the greater the bonding power, the better the bonding performance between warm mix asphalt and limestone aggregates.

(4) $$W_{\mathrm{as}}{=\gamma }_{a}\left(1+\cos\theta \right)=2\sqrt{\gamma_{a}^{d}\gamma_{s}^{d}}+2\sqrt{\gamma_{a}^{+}\gamma_{s}^{-}}+2\sqrt{\gamma_{a}^{-}\gamma_{s}^{+}}$$

Among them, $W_{as}$ is asphalt–aggregate adhesive work, $\gamma_{a}$ is asphalt surface energy, $\gamma_{s}$ is aggregate surface energy, θ  is contact angle between the asphalt and the aggregate, $\gamma_{a}^{d}$ and $\gamma_{s}^{d}$ are the dispersion components of asphalt and aggregate, and $\gamma_{a}^{p}$ and $\gamma_{s}^{p}$ are the polar components of asphalt and aggregate, respectively.

### 2.6. Contribution Rate Calculation Model

The double cumulative curve method was used to study the effects of ultraviolet radiation and oxygen on the adhesive performance indicators and functional group index of WMA. The input factors of the model include carbonyl group, sulfoxide group, adhesive work and adhesive work. Taking the influence on the change in adhesive work as an example, the calculation method of contribution rate is shown in Equations (5)–(10).

(5) $$\sum Q_{oxygen\;barrier}=k\sum P_{oxygen\;barrier}+b$$

(6) $$\sum Q_{light\;oxygen\;coupling}=k\sum P_{light\;oxygen\;coupling}+b\;$$

In the equation, $\sum Q_{oxygen\;barrier}$ is the cumulative calculated value of the adhesive work of oxygen resistant ultraviolet aging, $\sum Q_{light\;oxygen\;coupling}$ is the cumulative calculated value of light–oxygen-coupled UV aging adhesive work, $\sum P_{oxygen\;barrier}$ is the cumulant amount of adhesive work of oxygen-free ultraviolet aging, $\sum P_{light\;oxygen\;coupling}$ is the cumulant of the adhesive work of light–oxygen-coupled UV aging, and k and b are the linear regression equation parameters of the UV adhesive work curve in the process of oxygen-free UV aging.

(7) $$\bar{\nabla Q_{oxygen}}=\left(\sum Q_{light\;oxygen\;coupling}-\sum Q_{oxygen\;barrier}\right)/n$$

(8) $$\bar{\nabla Q}=\sum Q_{light\;oxygen\;coupling}/n$$

In the formula, $\bar{\nabla Q}$ is the average adhesive work under light–oxygen coupling conditions, $\bar{\nabla Q_{oxygen}}$ is the calculated average adhesive work under the influence of oxygen, and n is the aging time.

(9) $$\varepsilon_{oxygen}=\frac{\bar{\nabla Q_{oxygen}}}{\bar{\nabla Q}}\times 100\%$$

(10) $$\varepsilon_{ultraviolet}=1-\varepsilon_{oxygen}$$

In the equation, $\varepsilon_{oxygen}$ is the contribution rate of oxygen to the change in adhesive work, and $\varepsilon_{ultraviolet}\;$ is the contribution rate of ultraviolet radiation to changes in adhesive work.

### 2.7. Random Forest Model

Random forest has obvious advantages in processing multidimensional data and is one of the best classification algorithms at present. Firstly, it uses the bootstrap method to extract k samples from the original training sample set N. Secondly, the corresponding decision tree model is established for k samples. Finally, the k sample results obtained are voted on, and the final classification result is selected based on the principle of minority obeying majority [33]. The classification decision function is shown in Equation (11).

(11) $$H\left(x\right)=\arg\;max\;y\sum_{i=1}^{k}I\left[h_{i}\left(x\right)=Y\right]$$

In the formula, $H\left(x\right)\;$ is the combined classification model; $h_{i}$ is the decision classification model; Y is the output variable adhesive work, cohesive work, carbonyl index and sulfoxide index. Taking them as the corresponding characteristics, the importance of the characteristics is determined so as to achieve the purpose of classification. According to this analysis, random forest classification model is used. Random forest model is established according to the data, and the importance of characteristics is calculated.

## 3. Results and Discussion

### 3.1. Adhesive Performance Analysis Based on SFE

#### 3.1.1. Contact Angle

From Table 4, it can be seen that as the degree of aging increases, the contact angle between WMA and water gradually increases, indicating that aging improves the hydrophobicity of asphalt. Comparing the coupling of light and oxygen and the isolation of oxygen, it was found that the addition of oxygen increases the contact angle. In addition, the larger the contact angle, the more cos θ, the smaller the value, the weaker the wettability and the better the floatability. The smaller the contact angle, the cos θ, the larger the value, the stronger the wettability and the worse the floatability. It can be seen that the floatability of WMA will increase after UV aging.

#### 3.1.2. Adhesive Work

**Figure 6:**
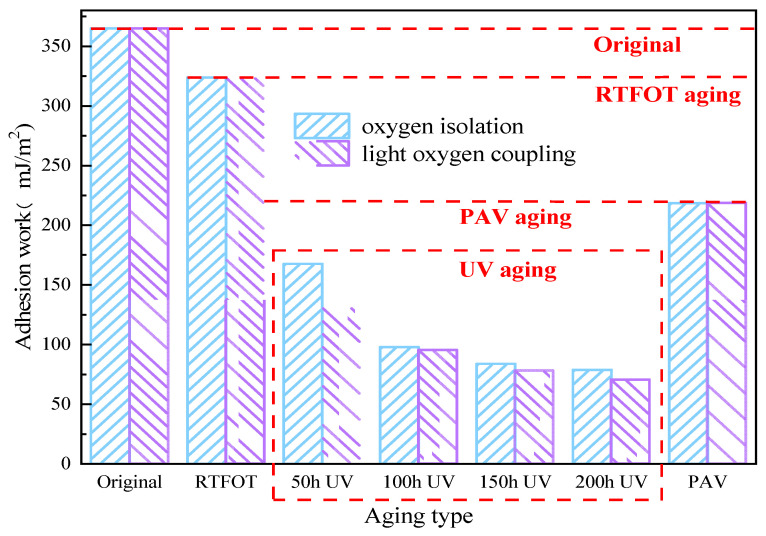
Adhesion work of WMA under different aging conditions.

The adhesive work reflects the bonding quality between aggregates and asphalt. A large adhesive strength indicates good adhesive performance and strong resistance to water damage. The adhesion between WMA and the limestone aggregate system was studied before and after UV aging. The experimental results are shown in Figure 6.

The results in Figure 6 show that the cohesive work of warm mix asphalt without aging is 365.03 mJ/m^2^. After RTFOT aging, it was 323.73 mJ/m^2^, a decrease of 11.3%. After aging, PAV was 218.51 mJ/m^2^, a decrease of 40.1%. After aging, it was 167.69 mJ/m^2^, 97.90 mJ/m^2^, 83.89 mJ/m^2^ and 78.86 mJ/m^2^, which are decreases of 54.1%, 73.2%, 77.0% and 78.4%, respectively. After light–oxygen-coupled aging, it was 130.96 mJ/m^2^, 95.25 mJ/m^2^, 77.86 mJ/m^2^ and 70.36 mJ/m^2^, which are decreases of 64.1%, 73.9%, 78.1% and 80.7%, respectively. Compared with light–oxygen-coupled aging and oxygen isolation aging, it decreased by 21.9%, 2.7%, 7.2% and 10.8%.

It can be seen from Figure 6 that in RTFOT aging, PAV aging and UV aging, the adhesive work of WMA showed a decreasing trend. Compared with RTFOT and PAV aging, UV aging makes the WMA decline more obviously. It shows that UV aging and thermo-oxidative aging have different effects on WMA, and UV aging has a greater impact on WMA adhesive work. UV aging reduces the WMA adhesive work, and when the UV aging time exceeds 150 h, the decreasing trend gradually becomes stable. Under the light–oxygen coupling condition, the WMA adhesive work is smaller. This shows that the presence of oxygen aggravates the reduction in WMA adhesive work, which reduces the water damage resistance of WMA. This may be due to the light components in WMA being more likely to react with the presence of oxygen. As a result, the hard components in WMA increase, and WMA becomes hard, which further reduces the bonding performance of WMA.

#### 3.1.3. Cohesive Work

**Figure 7:**
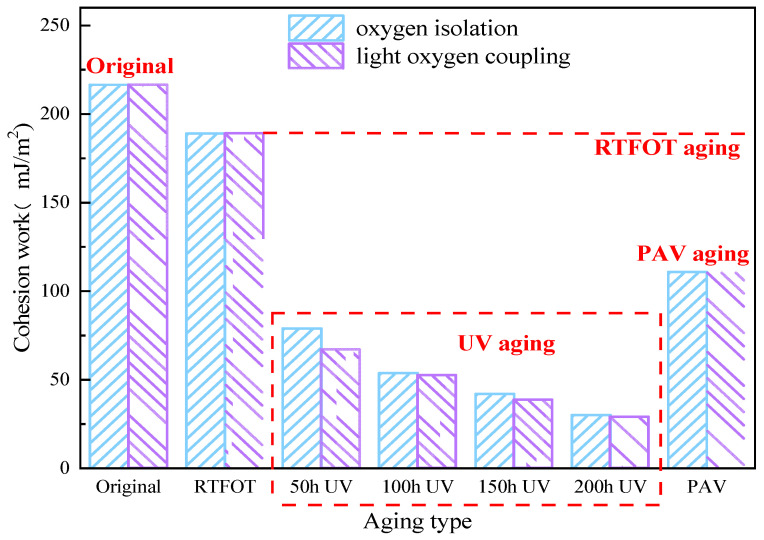
Conhesion work of WMA under different aging conditions.

Cohesive work refers to the energy consumed to overcome the interaction between asphalt molecules. The greater the cohesive work is, the better the cohesive property of asphalt is and the stronger the cracking resistance is. The cohesive work of WMA before and after aging in different ways and times is shown in Figure 7.

The results in Figure 7 show that the cohesive work of warm mix asphalt without aging is 216.52 mJ/m^2^. After RTFOT aging, it was 189.07 mJ/m^2^, a decrease of 12.6%. After aging, PAV was 110.79 mJ/m^2^, a decrease of 48.8%. After aging, it was 78.98 mJ/m^2^, 53.8 mJ/m^2^, 42 mJ/m^2^ and 30.2 mJ/m^2^, which are decreases of 63.5%, 75.2%, 80.6% and 86.1%, respectively. After light–oxygen-coupled aging, it was 69.1 mJ/m^2^, 52.5 mJ/m^2^, 38.56 mJ/m^2^ and 28.98 mJ/m^2^, which are decreases of 68.1%, 75.8%, 82.2% and 86.7%, respectively. Compared with light–oxygen-coupled aging and oxygen isolation aging, it decreased by 12.5%, 2.4%, 8.2% and 4.0%.

It can be seen that the cohesive work of warm mix asphalt shows a downward trend after aging. Compared with RTFOT, PAV and UV aging, the reduction in the cohesive work of warm mix asphalt after UV aging is the largest, and the reduction in RTFOT is the smallest. With the extension of UV aging time, the cohesive work decreased gradually. When UV aging is carried out for 50 h, oxygen has the greatest influence on the cohesive work of warm mix asphalt and then gradually becomes stable. In general, when there is oxygen, the ability of the anti-adhesive damage of warm mix asphalt after UV aging is the weakest. When the light–oxidation-coupled ultraviolet aging exceeds 150 h, the possibility of cohesive failure of warm mix asphalt is the largest, indicating that the water damage resistance is the worst at this time.

### 3.2. Functional Groups Analysis Based on FTIR

**Figure 8:**
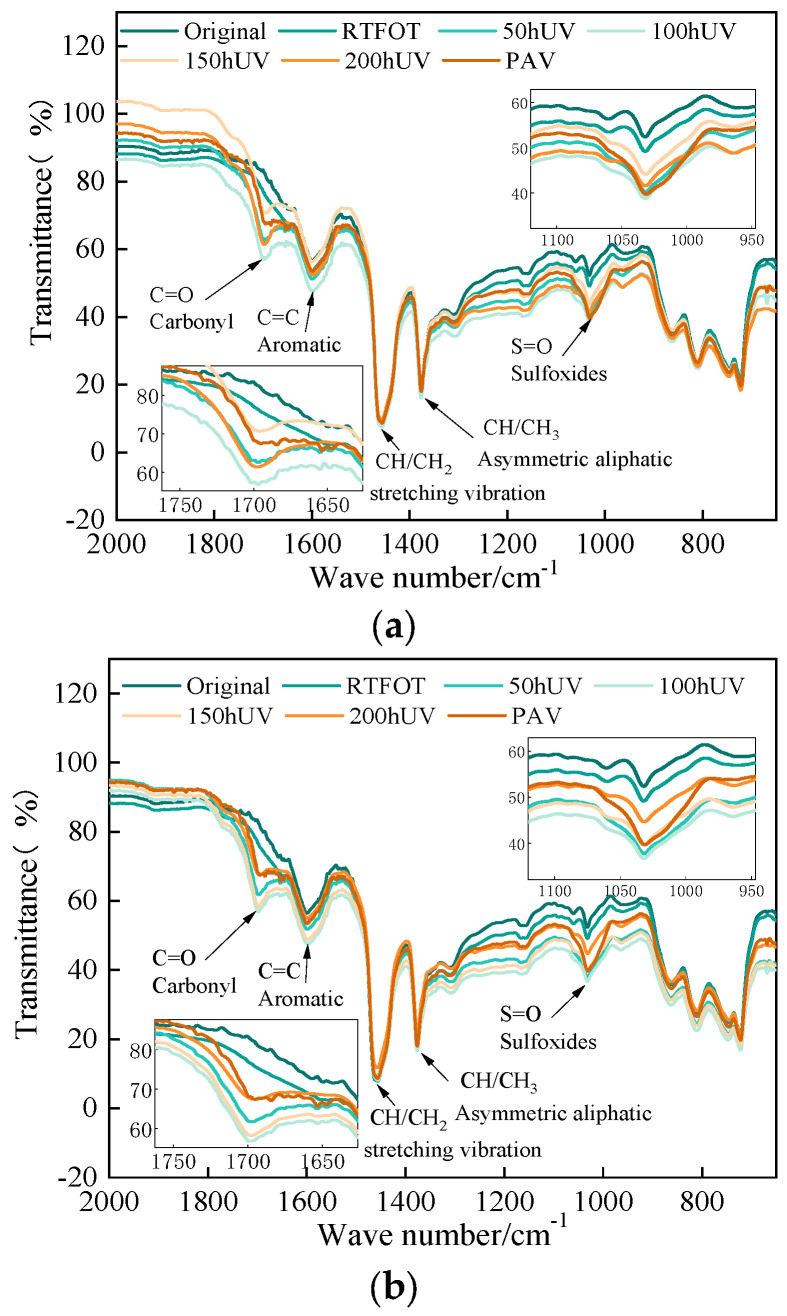
FTIR spectrum of WMA: (**a**) oxygen barrier; (**b**) light–oxygen coupling.

The carbonyl (C=O) absorption peak at 1700 cm^−1^ and the sulfoxide (S=O) stretching vibration peak at 1030 cm^−1^ of WMA increased significantly during oxygen barrier aging and light–oxygen-coupled UV aging. Comparing oxygen barrier aging and light–oxygen-coupled UV aging, it was found that the light–oxygen-coupled UV aging condition has a greater effect on these two absorption peaks than the oxygen barrier aging condition. The peak and area of the sulfoxide (S=O) functional group located at 1030 cm^−1^ increased slightly after short-term aging. However, after PAV aging and light–oxygen-coupled UV aging, the sulfoxide functional group changed significantly, indicating that a severe oxidation reaction occurred in the warm mix asphalt. FTIR spectrum of WMA is shown in Figure 8. The analysis of the causes showed that the sulfoxide group is a characteristic peak that characterizes the oxygen absorption aging of asphalt, and the increase in this peak indicates that the asphalt has undergone oxygen absorption aging. As a result, its properties will change significantly with the participation of oxygen. In addition, the change in functional groups after aging of PAV is comparable to the result of 50 h of UV aging, while the carbonyl peak of WMA stabilizes after 150 h of UV aging.

According to the changing law of each functional group index during the aging process of asphalt, it can reflect the changes in the chemical structure of asphalt before and after aging [34]. Usually, the carbonyl index and the sulfoxide index are chosen as the indicators to quantitatively characterize the changes in the chemical structure of warm mix asphalt during UV–oxygen coupling and oxygen barrier aging. The carbonyl index and sulfoxide index are calculated from the area ratio of the characteristic peaks in the infrared spectra to the peak region between 600 cm^−1^ and 2000 cm^−1^, which is calculated as follows:

(12) IC=O=A1738∑A600~2000

(13) IS=O=A1030∑A600~2000

**Figure 9:**
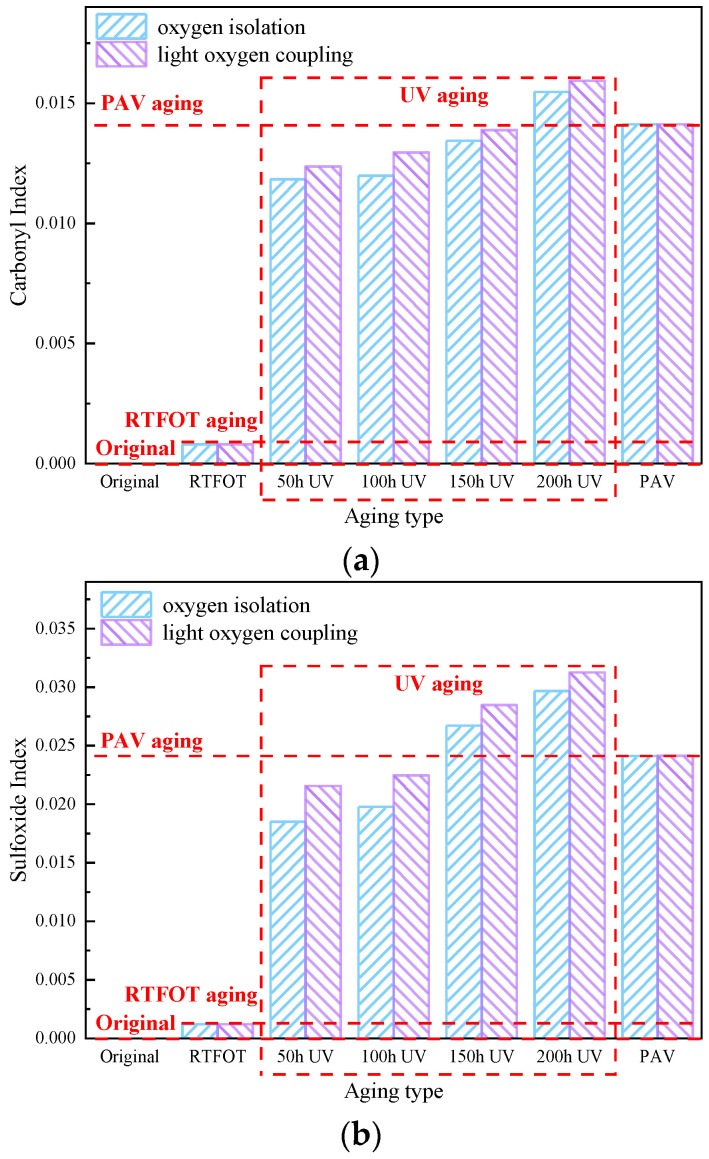
Functional group index of WMA: (**a**) carbonyl index; (**b**) sulfoxide index.

As can be seen in Figure 9, the carbonyl index gradually increases with aging time under oxygen barrier and light–oxygen conditions, indicating that warm mix asphalt undergoes a significant oxidation reaction under UV irradiation, and the degree of aging increases with UV aging time, gradual deepening. The trend of the carbonyl index of warm mix asphalt under light–oxygen-coupled UV aging is consistent with that under oxygen-isolating conditions, but the carbonyl index of warm mix asphalt under light–oxygen-coupled UV aging is significantly higher than that of warm mix asphalt under oxygen-isolating UV aging, indicating that there is the participation of oxygen, and the degree of aging of warm mix asphalt is more serious.

In addition, under oxygen-isolating UV aging conditions, the sulfenyl index of WMA increased rapidly with the extension of UV aging time, indicating that warm mix asphalt under UV irradiation undergoes a significant oxidation reaction, and the degree of aging increases with the increase in UV aging time. The aging time is gradually prolonged and deepened; the change trend of the sulfenyl group index of warm mix asphalt under light–oxygen-coupled UV aging is consistent with that under oxygen-isolating conditions, but the sulfenyl group index of warm mix asphalt under light–oxygen-coupled UV aging is significantly higher than that under oxygen-isolating conditions. The results of the sulfoxide group index of warm mix asphalt under oxygen UV aging conditions show that oxygen exacerbates the UV aging of warm mix asphalt. In addition, under different UV aging conditions, the carbonyl and sulfoxide group contents of warm mix asphalt increased gradually with the extension of UV aging time, but the growth rate of the carbonyl index was significantly higher than that of the sulfoxide group, suggesting that UV aging makes C=C oxidation more serious [35,36].

### 3.3. Correlation Analysis

**Figure 10:**
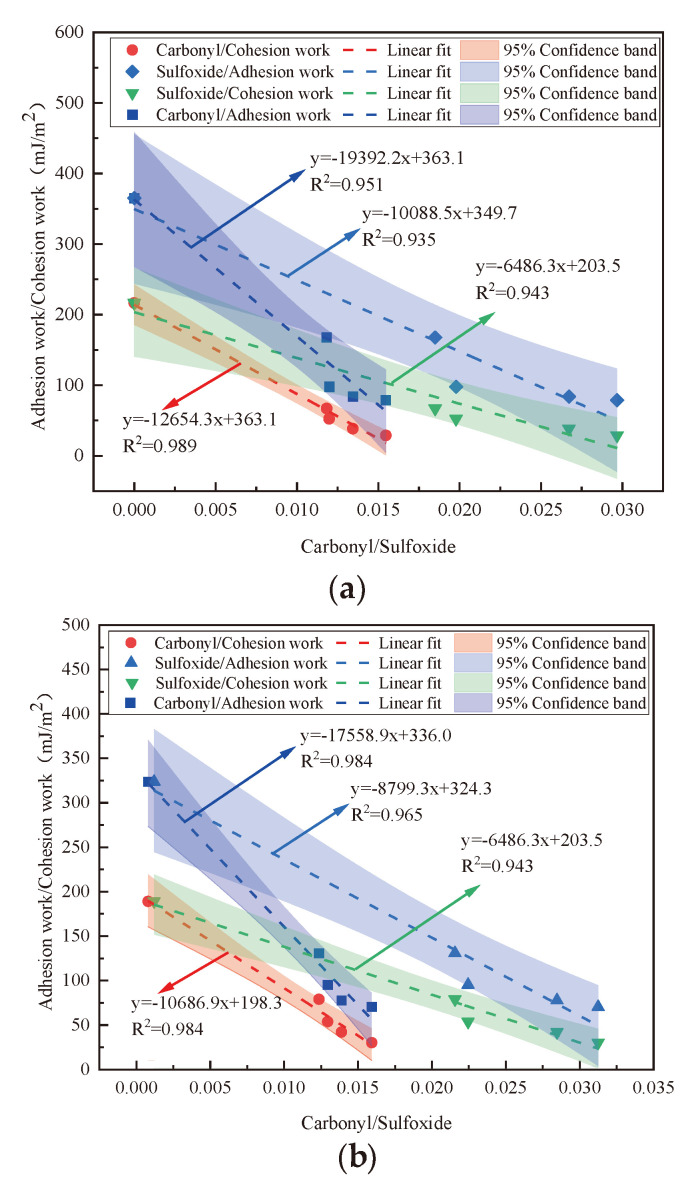
Confidence interval: (**a**) oxygen barrier; (**b**) light–oxygen coupling.

The correlation regression analysis of surface roughness and adhesive work of WMA under UV aging condition was carried out by using Origin software 8.5, and the results are shown in Figure 10.

The figure shows the linear fitting relationship between the adhesive work and the roughness in the two environments. The correlation coefficients between surface roughness and adhesive work under light–oxygen coupling and single UV aging conditions are 0.9114 and 0.9067, respectively. All points fall within the 95% confidence band, indicating that the WMA roughness and adhesive work have an excellent fitting effect. In addition, under the action of light–oxygen coupling, the fitting points of adhesive work and roughness are relatively uniformly dispersed and closer to the fitting line [39,40]. While the fitting points are relatively aggregated under the action of the oxygen barrier and deviate from the fitting line, the Pearson index of the fitting curve under the action of light–oxygen coupling is larger, indicating a better fit.

### 3.4. Significant Difference Analysis

A significant difference is an evaluation of the difference in data. SPSS 19 software was used to compare the difference between the oxygen barrier and light–oxygen-coupled UV aging. The results are shown in Table 5. If “F > F crit”, there is a significant difference; if “F < F crit”, there is no significant difference. Combined with *p*-value, if “0.01 < *p*-value < 0.05”, it means that the difference is significant, and if “*p*-value < 0.01”, it means that the difference is extremely significant.

Comparing the different sources of difference, F was significantly greater than the F-specific volume under the four sources of difference. This indicates that there are significant differences in the performance parameters of WMA due to the coupling of oxygen barrier and light–oxidative aging. The *p*-value results show that the *p*-values of adhesion and cohesion functions are 0.0191 and 0.006786, respectively, which are significantly different from each other. However, the *p*-values for carbonyl and sulfoxide indices were 0.000763 and 0.000613, respectively. Both results were significantly less than 0.01, indicating that the differences between oxygen barrier and light–oxygen-coupled UV aging were highly significant. In addition, the *p*-values for the adhesion and cohesion functions of WMA after oxygen barrier and light–oxygen-coupled UV aging were 0.0191 and 0.006786, respectively. Combined with the *p*-value results for the carbonyl group and the sulfoxide group, it was concluded that the significant differences between the oxygen barrier and light–oxygen-coupled adhesives may be due to the changes in the cohesion of WMA. The main reason for the change in the cohesion of WMA after UV aging is the presence of oxygen. It can be seen that there is a significant difference between the effects of oxygen barrier and light–oxygen-coupled UV aging on WMA, and it is necessary to conduct an in-depth study on the mechanism of oxygen barrier UV aging of WMA at a later stage.

### 3.5. Contribution Rate of Ultraviolet and Oxygen

To investigate the contribution of oxygen and ultraviolet radiation to the adhesive index of WMA under light–oxygen-coupled aging conditions, according to Equations (5)–(10), the contribution rates of ultraviolet and oxygen to the adhesive index of WMA were calculated, as shown in Table 6.

The results in Table 6 show that the contribution rates of ultraviolet radiation to the carbonyl index, sulfoxide index, adhesive work and adhesive work of WMA are 78.6%, 79.0%, 79.2% and 82.8%, respectively. The contribution rates of oxygen to the carbonyl index, sulfoxide index, adhesive work and adhesive work of WMA are 21.4%, 21.0%, 20.8% and 18.2%, respectively. From this, it can be seen that the contribution rate of ultraviolet radiation in the UV aging process of WMA is about 80%, and the contribution rate of oxygen is about 20%.

### 3.6. Importance of Independent Variables

**Figure 11:**
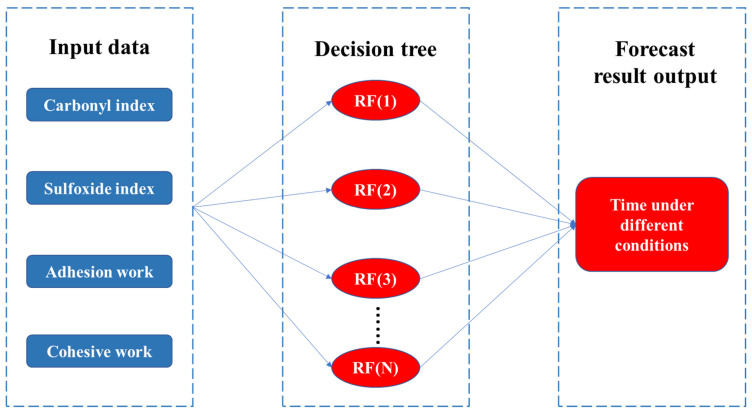
RBF neural network model.

**Figure 12:**
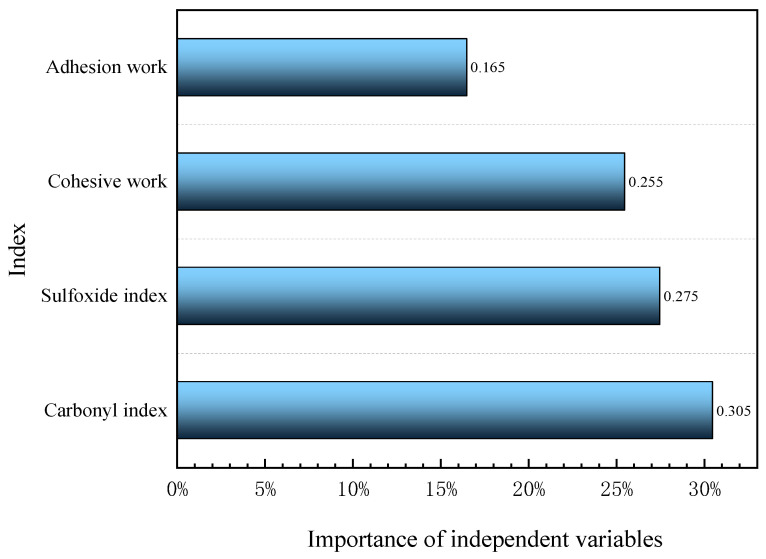
Importance of independent variables.

To further analyze the accurate contribution rate of UV and oxygen in the UV aging process of WMA, the adhesive performance evaluation parameters of WMA were optimized using the WMA adhesive performance index as the output layer of the RBF neural network model. Firstly, an RBF neural network model was established using SPSS software. Then, the WMA adhesive performance index was set as the dependent variable in the output layer. The softmax activation function was used to put the normalized data into the hidden layer, as shown in Figure 11. The importance analysis of independent variables is shown in Figure 12.

**Figure 13:**
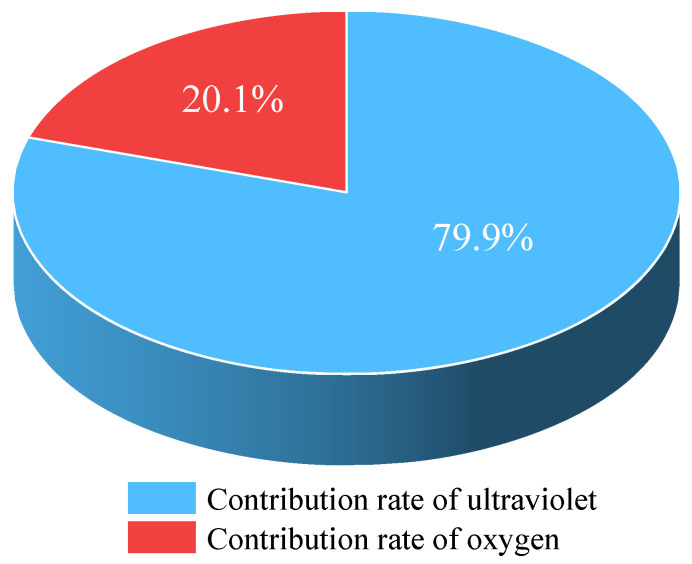
Contribution rate of ultraviolet and oxygen.

The adhesive performance evaluation parameters of WMA were optimized through RBF neural network model analysis. The results in Figure 12 indicate that the importance of adhesive work, cohesive work, sulfoxide index and carbonyl index is 0.165, 0.255, 0.275 and 0.305, respectively. The carbonyl index has the greatest impact on the adhesive performance of WMA during UV aging, followed by the sulfoxide index and adhesive work, with adhesive work having the lowest importance. In addition, based on the contribution rate in Table 6 and the importance index in Figure 12, this article calculates the contribution rate of ultraviolet and oxygen to the aging degree of WMA during the UV aging process according to Equations (14) and (15), as shown in Figure 13.

(14) $$\varepsilon_{UV}=\varepsilon_{UV\left(CI\right)}\cdot k_{UV\left(CI\right)}+\varepsilon_{UV\left(SI\right)}\cdot k_{UV\left(SI\right)}+\varepsilon_{UV\left(AW\right)}\cdot k_{UV\left(AW\right)}+\varepsilon_{UV\left(CW\right)}\cdot k_{UV\left(CW\right)}$$

(15) $$\varepsilon_{O_{2}}=1-\varepsilon_{UV}$$

In the equations, $\varepsilon_{UV}$ is the contribution rate of UV to ultraviolet radiation, $\varepsilon_{UV\left(CI\right)}$ is the contribution rate of ultraviolet radiation to the carbonyl index, $k_{UV\left(CI\right)}$ is the importance coefficient of ultraviolet radiation in the carbonyl index, $\varepsilon_{UV\left(SI\right)}$ is the contribution rate of ultraviolet radiation to the sulfoxide index, $k_{UV\left(SI\right)}$ is the importance coefficient of ultraviolet radiation in the sulfoxide index, $\varepsilon_{UV\left(AW\right)}$ is the contribution rate of ultraviolet radiation to adhesive work, $k_{UV\left(AW\right)}$ is the importance coefficient of ultraviolet radiation in adhesive work, $\varepsilon_{UV\left(CW\right)}$ is the contribution rate of ultraviolet radiation to the cohesive work, and $k_{UV\left(CW\right)}$ is the importance coefficient of ultraviolet radiation in adhesive work.

## 4. Conclusions

In this research, a monolithic UV aging and light–oxygen coupling device was developed, and a UV aging test method was established. The adhesive properties and functional group changes in WMA under oxygen barrier and photo-oxygen coupling conditions were analyzed using surface free energy and infrared spectroscopy. The relationship between different indexes was established, and the differences between oxygen barrier and light–oxygen-coupled UV aging were comparatively analyzed. Based on the results discussed above, the following conclusions can be summarized:

(1) RTFOT, PAV and UV aging all cause deterioration of bonding properties and significant changes in the functional group index of WMA, with the main chemical changes occurring during the aging process. Among them, UV aging has the greatest effect, followed by PAV aging, and RTFOT aging has the least effect.
(2) The increase in UV aging time leads to the hardening of WMA and thus to a decrease in the bonding and cohesion functions. The carbonyl index and sulfoxide index increased with the increase in UV aging time, and the main WMA mainly absorbed oxygen with asphalt during the photo-oxidative coupling process and accelerated its aging. After 150 h of UV aging, the bonding performance index and functional group index gradually tend to stabilize.
(3) The correlation coefficients for photo-oxidative coupling UV aging are higher than those for oxygen barrier UV aging. Photo-oxidative coupling and oxygen-free UV aging have significant effects on the adhesion and cohesive work of WMA. The effects of the carboxyl index and sulfoxide index were extremely significant.
(4) There is a significant difference in the cohesion function of WMA after UV aging. The main reason for this significant difference may be the change in cohesion caused by molecular reactions within WMA due to the presence of oxygen.
(5) The random forest model shows that during the light–oxygen-coupled UV aging process of warm mix asphalt, the contribution rate of UV is 79.9%, and the contribution rate of oxygen is 20.1%.

## Figures and Tables

**Table 1:** Indoor and outdoor ultraviolet radiation time conversion table.

Indoor UV aging time/h	50	100	150	200
Outdoor UV aging time/month	1	2	3	4

**Table 2:** Properties index of WMA.

Items		Units	Requirement	Result
25 °C penetration/(100 g, 5 s)		0.1 mm	80~100	86.2
Softening point/(R&B)		°C	≥42	47.3
10 °C ductility		cm	≥20	25.2
RTFOT (163 °C, 85 min)	Mass loss	%	≤±0.8	0.04
	Penetration ratio	%	≥57	67.2
	Ductility/10 °C	cm	≥8	11.4

**Table 3:** Surface free energy parameters of three test liquids (25 °C) mJ/m^2^.

Probe Liquids	$\gamma_{L}$	$\gamma_{L}^{d}$	$\gamma_{L}^{p}$	$\gamma_{L}^{+}$	$\gamma_{L}^{-}$
Distilled water	72.8	21.8	51.0	25.50	25.5
Glycerol	64.0	34.0	30.0	3.92	57.4
Formamide	58.0	38.0	19.0	2.28	39.6

**Table 4:** Contact angle of WMA before and after UV aging.

Test Liquids	Contact Angle (°)							
	Original	RTFOT	Oxygen (Yes or No)	50 h UV	100 h UV	150 h UV	200 h UV	PAV
Distilled water	99.42	97.72	No	101.96	103.97	103.15	99.26	104.96
			Yes	99.97	98.11	99.68	101.18	
Glycerol	103.46	100.29	No	95.89	94.82	94.04	92.42	101.04
			Yes	97.03	97.32	94.53	95.11	
Formamide	80.87	79.85	No	85.13	85.48	86.89	88.71	85.53
			Yes	81.50	85.68	84.75	85.31	

**Table 5:** Significant difference analysis under light–oxygen coupling and oxygen barrier conditions.

Source of Difference	SS	df	MS	F-Value	*p*-Value	F Crit
Adhesive work	6909.03	3	2303.01	18.71219	0.0191	9.276628
Cohesive work	3.22895	3	1.076317	11.65896	0.006786	9.276628
Carbonyl index	1.58 × 10^−5^	3	5.27 × 10^−6^	169.1818	0.000763	9.276628
Sulfoxide index	0.000152	3	5.08 × 10^−5^	196.085	0.000613	9.276628

**Table 6:** Contribution rates of ultraviolet and oxygen.

Index	Contribution Rate	
	Ultraviolet	Oxygen
Carbonyl index	78.6%	21.4%
Sulfoxide index	79.0%	21.0%
Adhesive work	79.2%	20.8%
Cohesive work	82.8%	18.2%

## Data Availability

The original contributions presented in this study are included in the article. Further inquiries can be directed to the corresponding authors.
